# Total Colonic Decompression After Colonoscopy for the Prevention of Post-colonoscopy Abdominal Pain: A Systematic Review and Meta-Analysis of Randomized Controlled Trials

**DOI:** 10.7759/cureus.117805

**Published:** 2026-10-12

**Authors:** Mohammed Karkaman, Basel Alasmari, Fatimah Ghaidan A. Almuawi, Abdulaziz Mohammed Aldossari, Rayan Mahdi M. Al Awn, Shouq Mohammed Alshahrani, Bushra Mohammed Alshahrani, Saad Mansour Mohammed Alqahtani, Elaf Ali Alamri, Safar Abdullah Safar Al-Shahrani, Bayan Saad K. Alharthi, Afnan Mohammed N. Al-Shahrani, Ayed M. Al-Qahtani, Abdullah Alhussein A. Almonawar, Khalid Mohammed Algarni

**Affiliations:** 1 School of Medicine, University of Bisha, Bisha, SAU; 2 Medicine and Surgery, University of Bisha, Bisha, SAU; 3 College of Medicine, University of Bisha, Bisha, SAU

**Keywords:** air aspiration, colonoscopy, discomfort, post-colonoscopy abdominal pain, total colonic decompression

## Abstract

Post-colonoscopy abdominal pain and bloating are common adverse events associated with residual intraluminal air. Total colonic decompression (TCD), involving reintubation of the colonoscope to the cecum followed by active air aspiration during withdrawal, has been proposed to alleviate these symptoms; however, evidence regarding its clinical effectiveness remains inconsistent. This systematic review and meta-analysis evaluated the efficacy and safety of TCD compared with standard colonoscopy withdrawal. PubMed, Scopus, and Web of Science were systematically searched from inception through September 2026 for randomized controlled trials evaluating TCD versus standard withdrawal in adults undergoing colonoscopy with room-air insufflation. The primary outcomes were the incidence and severity of early and 24-hour post-colonoscopy abdominal pain. Risk ratios and mean differences with 95% confidence intervals were pooled using random-effects models. The protocol was registered in PROSPERO (CRD420261505215). Four randomized controlled trials involving 1,037 participants were included. TCD reduced the incidence of early post-colonoscopy abdominal pain compared with standard withdrawal, whereas no statistically significant reduction was observed in early pain severity. Similarly, TCD did not demonstrate a statistically significant reduction in either the incidence or severity of post-colonoscopy abdominal pain at 24 hours. Overall, total colonic decompression may reduce the occurrence of early post-colonoscopy abdominal pain but does not appear to provide a sustained reduction in pain severity. Further randomized controlled trials using standardized decompression techniques and consistent outcome assessment are warranted to clarify its clinical utility.

## Introduction and background

Colonoscopy is widely recognized as the most reliable method for detecting and monitoring colorectal cancer [1]. It is highly recommended by numerous national and global organizations as an early detection and management strategy for subsequent diagnosis and follow-up treatment after diagnosis [2,3].

Although colonoscopy is generally well tolerated, a subset of patients perceives the procedure as uncomfortable and painful [4]. 

Abdominal bloating and discomfort are the most common complaints reported by patients after colonoscopy, often contributing to post-procedural morbidity and work absenteeism [5]. The acute distress is mainly caused by intraluminal air distension, which results from continuous insufflation during the procedure and causes many patients to experience physiological difficulties in expelling residual room air during their immediate recovery [6]. 

Consequently, active aspiration of residual intraluminal air has been proposed to alleviate bowel distension and mitigate the incidence of post-colonoscopy abdominal pain (PCAP) [7]. Total colonic decompression (TCD), defined as reintubating the colonoscope back to the cecum upon completion of standard mucosal examination, followed by targeted air aspiration during a secondary scope withdrawal, represents an intraprocedural strategy designed for this purpose [8].

Several randomized controlled trials have investigated the effect of total colonic decompression on post-colonoscopy abdominal pain, with inconsistent findings. Lee et al. and Park et al. reported that total colonic decompression reduced post-colonoscopy discomfort and abdominal pain, respectively [4,9]. Similarly, Sur et al. reported lower pain scores with more proximal reinsertion and air decompression [10]. In contrast, Aslan et al. found no statistically significant reduction in post-colonoscopy abdominal pain with total colonic decompression [8]. These conflicting findings create uncertainty regarding the clinical effectiveness of total colonic decompression and provide a rationale for synthesizing the available randomized evidence.

This systematic review and meta-analysis aimed to evaluate the effect of total colonic decompression after colonoscopy on post-colonoscopy abdominal pain compared with standard colonoscopy withdrawal without routine air decompression.

## Review

Methods 

Protocol and Registration

This systematic review and meta-analysis was carried out in accordance with PRISMA guidelines [11] and the methodological framework outlined in the Cochrane Handbook for Systematic Reviews of Interventions [12]. The review protocol was registered on PROSPERO (CRD420261505215).

Eligibility Criteria

Studies were eligible for inclusion if they were randomized controlled trials (RCTs) evaluating adult patients undergoing elective colonoscopy under room air insufflation who received total colonic decompression (reintubation to the cecum with active air aspiration during withdrawal) compared with standard colonoscopy withdrawal without routine air decompression. Studies conducted in any clinical, outpatient, or surgical endoscopy setting were eligible.

We excluded non-randomized studies, observational studies, case reports, case series, review articles, editorials, conference abstracts lacking full data, studies using insufflation exclusively, and studies evaluating non-total or non-cecal decompression techniques.

*Literature Search* 

A comprehensive systematic search was conducted across PubMed, Scopus, and Web of Science from inception to September 2026, without language restrictions. The same search strategy was applied across all three databases using the following terms: ((colonoscopy OR colonoscopic OR colonoscopies) AND (decompression OR decompress* OR "air aspiration" OR "gas aspiration" OR "air removal" OR "gas removal" OR "gas evacuation")). The final search was conducted in September 2026.

Study Selection

All retrieved citations were imported into EndNote for duplicate removal. Independent reviewers screened titles and abstracts against the eligibility criteria using Rayyan software [13]. Full-text articles of potentially eligible studies were subsequently evaluated independently by two authors. Any discrepancies were resolved through discussion or consultation with a third reviewer.

Data Extraction

Data were extracted independently by two reviewers using a standardized extraction form. Extracted variables included: Study characteristics - first author, publication year, country, sample size, and study design; Population and procedural variables - patient age, sex, body mass index (BMI), history of abdominal surgery or irritable bowel syndrome (IBS); Outcome measures - early pain severity (visual analog scale (VAS) [14]), late pain severity (at 24-48 h), and incidence of post-colonoscopy pain or discomfort.

Measures of Treatment Effect

The included studies assessed the efficacy and safety of total colonic decompression using clinical, procedural, and patient-reported outcomes. Primary efficacy outcomes focused on post-colonoscopy abdominal pain and discomfort. Pain severity was evaluated immediately or within 30-60 minutes post-procedure and at 24-48 hours post-procedure using validated continuous scales, specifically the 10-point VAS. The incidence of early and late post-colonoscopy abdominal pain was recorded as dichotomous outcomes.

Risk-of-Bias Assessment

The risk of bias for each included RCT was independently assessed using the Cochrane Risk of Bias tool for randomized trials (RoB 2) [15]. The tool evaluates five domains: bias arising from the randomization process, bias due to deviations from intended interventions, bias due to missing outcome data, bias in measurement of the outcome, and bias in selection of the reported result. Disagreements were resolved by consensus.

Data Synthesis and Analysis

All statistical analyses were performed with the Review Manager statistical software package (Version 5.4.1). Continuous pooled outcomes were reported as mean differences (MD) or standardized mean differences (SMD) and a 95% confidence interval (CI). To assess heterogeneity, we visually inspected the forest plot and tested the chi-squared distribution of the Cochrane Q statistic (a chi-square p-value <0.1 indicating significant heterogeneity) and the I2 statistic (>50% indicating considerable heterogeneity). For the meta-analytic effect sizes, a p-value <0.05 was considered statistically significant. We could not assess publication bias using the funnel plot and Egger’s test because the number of studies for each analysis model was less than 10 [16]

Results 

Literature Search Results

A total of 1560 records were identified through the literature search. Of these, 1056 were removed as duplicates, 504 records were screened for title and abstracts, and 490 were excluded as irrelevant or ineligible publication types.

Fourteen records were eligible for full‐text screening. Of them, four articles were included in qualitative and quantitative synthesis. The PRISMA 2020 flow diagram of the study selection process is shown in Figure 1.

**Figure 1:**
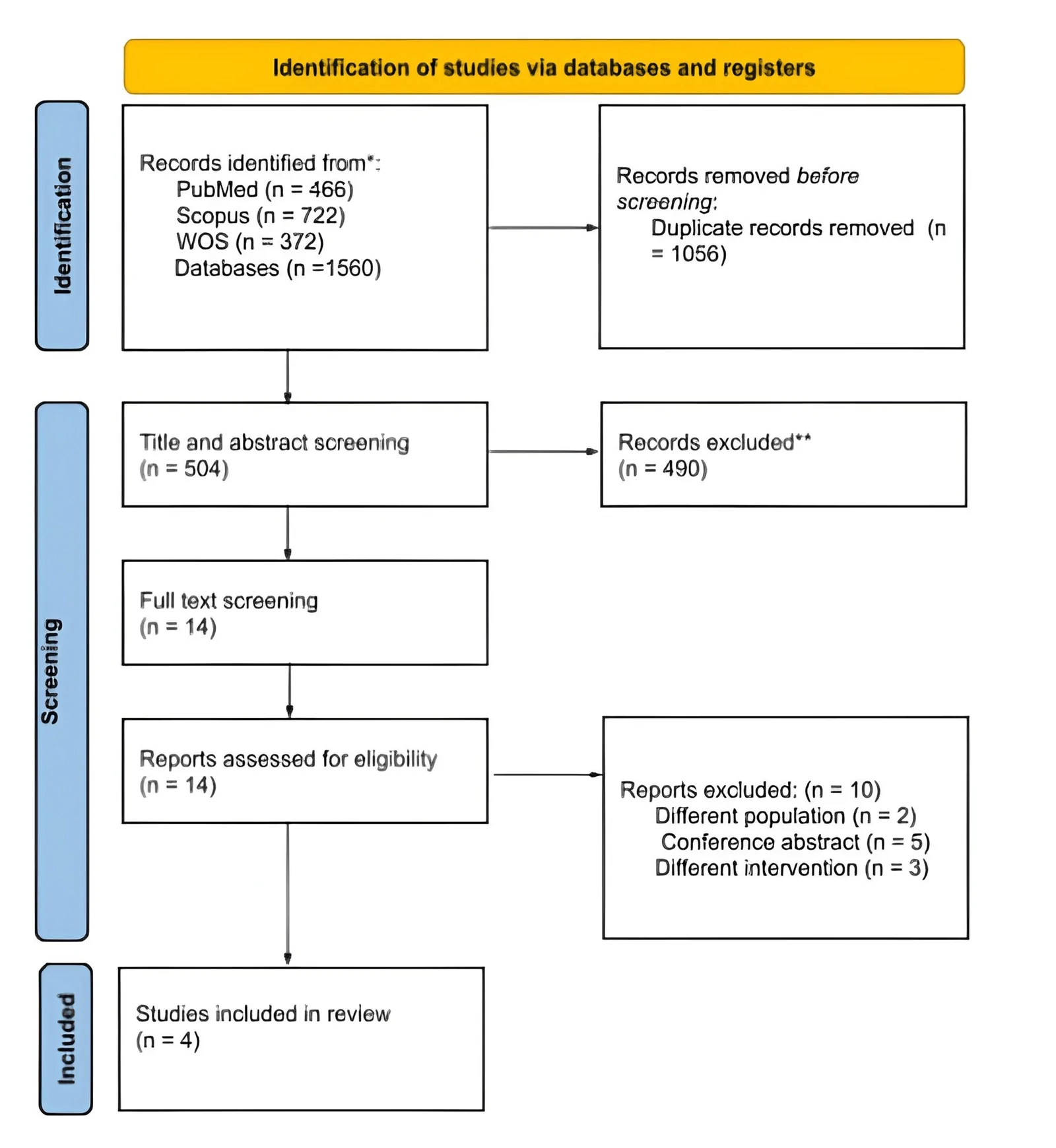
PRISMA 2020 flow diagram of study identification, screening, and inclusion The diagram summarizes study identification, screening, eligibility assessment, and inclusion in the systematic review, adapted from the PRISMA 2020 statement [11]

Characteristics of the Included Studies

The four included RCTs (Lee et al., 2001; Park et al., 2016; Sur et al., 2016; Aslan et al., 2026) were published between 2001 and 2026, comprising a total of 1,037 adult patients undergoing elective colonoscopy under conscious sedation. Across all trials, 399 participants received total colonic decompression (re-intubation of the colonoscope to the cecum with active intraluminal air aspiration during scope withdrawal), while 393 participants served as controls (standard scope withdrawal without routine air decompression) [9,4,10,8]. The remaining participants in Sur et al. were allocated to parallel segmental decompression subgroups [10].

The included studies were conducted in the United States (Lee et al.), South Korea (Park et al.; Sur et al.), and Turkey (Aslan et al.) [8,4,10,9]. The mean patient age across treatment arms ranged from 47.8 to 51.5 years, and the mean body mass index (BMI) ranged from 24.2 to 26.7 (kg/m2). Room air was uniformly used for colonic insufflation across all studies. A summary of the included studies and the characteristics of their populations are shown in Tables 1, 2.

**Table 1:** Summary of included studies PCAP, post-colonoscopy abdominal pain; VAS, visual analog scale

Study ID	Study Design	Location	Sample Size	Year	Population	Intervention	Comparator	Follow-up Duration	Outcome Measures	Key Findings
Aslan 2026 [8]	Double blind, prospective, and randomized controlled trial.	Turkey	218	2023 - 2024	Patients aged 18 years or older referred for outpatient screening/check-up colonoscopy	Total colonic decompression	No decompression	24 hours post-procedure	Pain severity, VAS score, Vital signs	Total colonic decompression fails to reduce post-colonoscopy abdominal pain and instead is linked to increased propofol requirements, which subsequently results in hypoxic events.
Lee 2001 [9]	Prospective, randomized controlled trial.	USA	126	1999	Outpatients undergoing elective colonoscopy performed by an attending endoscopist	Total colonic decompression	No decompression	24–48 hours post-procedure	Prevalence and severity of post-procedural bloating and abdominal pain, procedural duration	Total colonic decompression does not produce a significant reduction in post-colonoscopy bloating.
Park 2016 [4]	Prospective, randomized, double-blind controlled trial.	South Korea	288	2013	Asymptomatic adult outpatients 18 years or older undergoing elective screening, surveillance, or check-up colonoscopy	Total colonic decompression	No decompression	24 hours post-procedure	Incidence and severity of post-colonoscopy abdominal pain (PCAP), vital signs, procedural duration	Total colonic decompression after colonoscopy is beneficial, reducing post-colonoscopy abdominal pain without introducing additional complications.
Sur 2016 [10]	Prospective, randomized controlled trial (5-arm parallel design).	South Korea	405	2014 – 2015	Adults aged 21 years or older undergoing health check-up colonoscopy	Colonic decompression with specified anatomical locations	No decompression	24 hours post-procedure	Post-colonoscopy abdominal pain intensity,	Reinserting the scope to perform air decompression is an effective strategy for reducing post-colonoscopy abdominal pain, and evacuating air as the scope approaches the hepatic flexure on reinsertion appears to be the most effective approach for minimizing this pain.

**Table 2:** Baseline characteristics

Study ID	Group Name	Number	Age	Gender (Male), n (%)	BMI (kg/m2) (mean±SD)	Medical History/Comorbidities n (%)
Aslan 2026 [8]	Decompression	114	49.05 ± 11.78	56 (49.1)	26.66 ± 4.46	Comorbidities: 33 (28.9%); IBS: 22 (19.3%); Prior Surgery: 24 (21.1%)
	Control	104	51.47 ± 10.82	53 (51)	26.49 ± 4.07	Comorbidities: 37 (35.6%); IBS: 22 (21.2%); Prior Surgery: 24 (23.1%)
Lee 2001 [9]	Decompression	65	53 (43 - 63)	30 (46.2%)	N/A	Prior Pelvic Surgery: 16 (25.0%); Diverticulosis: 23 (35.0%)
	Control	61	55(43 - 65)	29 (47.5%)	N/A	Prior Pelvic Surgery: 16 (26.2%); Diverticulosis: 20 (33.0%)
Park 2016 [4]	Decompression	143	47.86± 10.89	77 (53.8)	24.20± 3.30	IBS: 14 (9.8%); Prior Surgery: 45 (31.5%)
	Control	145	50.19± 10.75	83 (57.2)	24.57± 3.08	IBS: 11 (7.6%); Prior Surgery: 47 (32.4%)
Sur 2016 [10]	Group 1 (Distal S-Colon)	77	47.60±10.22	43 (55.8)	24.20±2.98	IBS: 6 (7.8%); Prior Abdominal Surgery: 1 (1.3%)
	Group 2 (Splenic Flexure)	87	49.74±9.08	44 (50.6)	24.26±2.47	IBS: 4 (4.6%); Prior Abdominal Surgery: 1 (1.1%)
	Group 3 (Hepatic Flexure)	81	46.95±9.35	53 (65.4)	24.70±3.75	IBS: 1 (1.2%); Prior Abdominal Surgery: 1 (1.2%)
	Group 4 (Cecum)	77	47.55±9.68	52 (67.5)	24.08±3.03	IBS: 3 (3.9%); Prior Abdominal Surgery: 4 (5.2%)
	Group 0 (Control)	83	46.88±10.52	55 (66.3)	24.19±3.80	IBS: 2 (2.4%); Prior Abdominal Surgery: 5 (6.0%)

Risk-of-Bias Assessment

Risk of bias was assessed for all four included RCTs using the Cochrane RoB2 tool across five domains (Figures 2, 3). Of the five RoB-2 domains, risk of bias arising from the randomization process was rated high in Lee et al. and low in the remaining three trials (Aslan et al., Park et al., and Sur et al.), giving an overall distribution of 75% low risk and 25% high risk for this domain [8,4,10]. Bias due to deviations from intended interventions and bias due to missing outcome data were both judged low risk across all four included trials. Bias in measurement of the outcome was rated low risk in Aslan et al., Lee et al., and Sur et al., and as having some concerns in Park et al., reflecting the inherent difficulty of blinding self-reported pain outcomes to the withdrawal technique received [8,9,10,4]. Bias in the selection of the reported result was judged low risk in Aslan et al. and Lee et al., and as having some concerns in Park et al. and Sur et al. [8,9,4,10]. Taking all five domains together, the overall risk-of-bias judgment was low in Aslan et al., high in Lee et al. (driven by the randomization-process domain), and raised some concerns in both Park et al. and Sur et al. (driven predominantly by the outcome-measurement and outcome-selection domains). Across the four trials, the overall risk of bias was therefore low in 25%, raised some concerns in 50%, and was high in 25% of the included studies [8,9,4,10].

**Figure 2:**
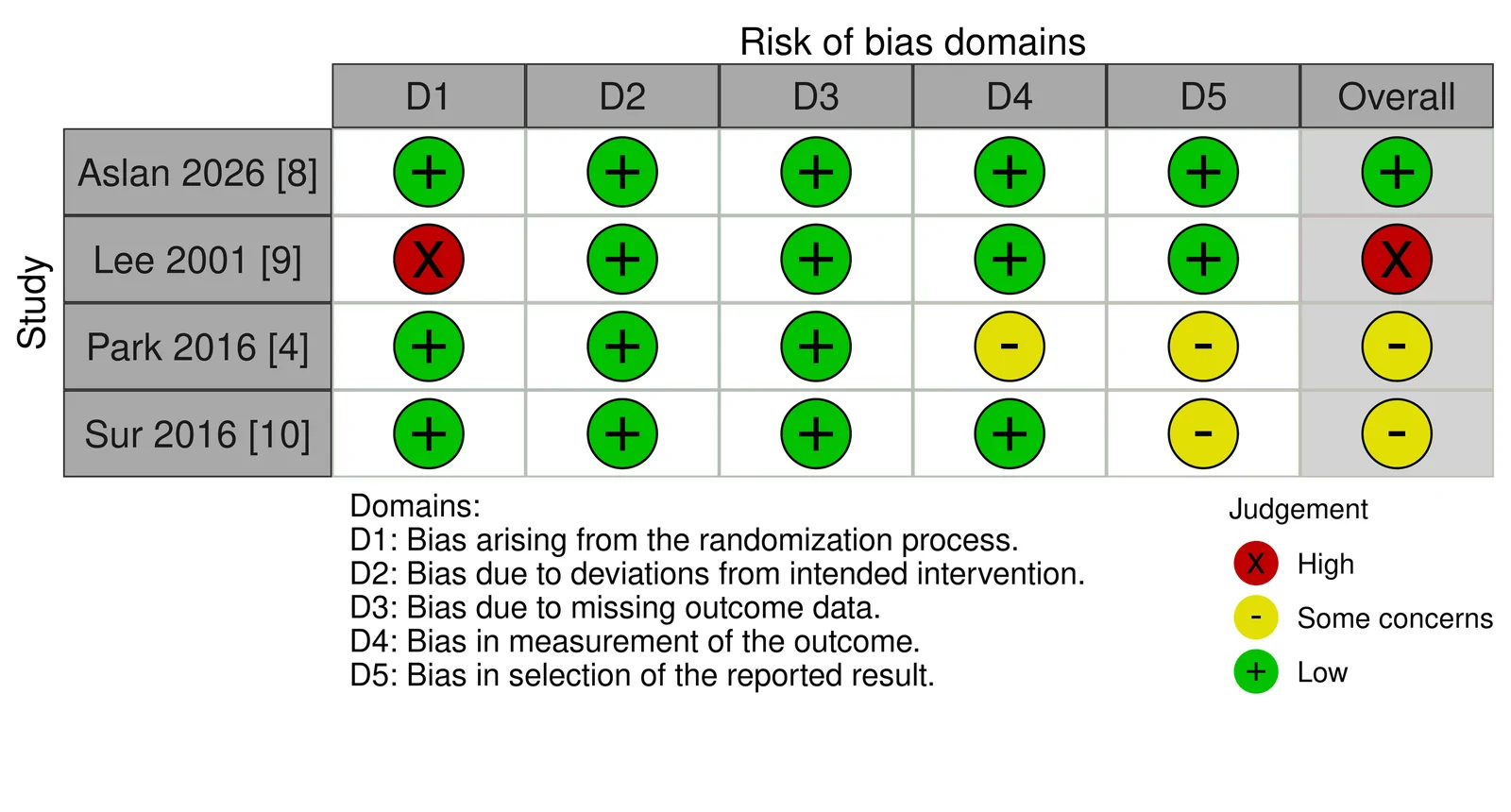
Risk-of-bias assessment of the included randomized controlled trials using the Cochrane Risk-of-Bias 2 tool D1, bias arising from the randomization process; D2, bias due to deviations from intended interventions; D3, bias due to missing outcome data; D4, bias in measurement of the outcome; D5, bias in selection of the reported result. Green indicates low risk of bias, yellow indicates some concerns, and red indicates high risk of bias. Risk of bias was assessed using the Cochrane Risk of Bias 2 (RoB 2) tool [15].

**Figure 3:**
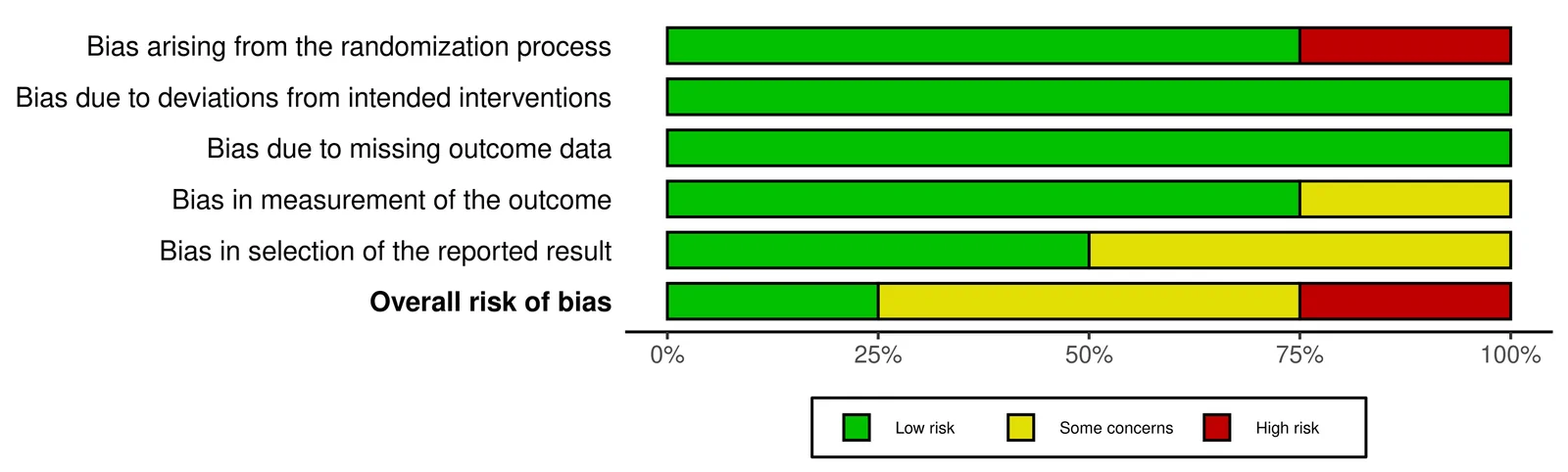
Bar plot illustrating the distribution of Risk-of-Bias 2 (RoB 2) risk-of-bias judgments across the included trials

Efficacy Outcome

Incidence of early post-colonoscopy pain: Total colonic decompression was associated with a significantly lower risk of early post-colonoscopy pain compared with control (RR 0.50, 95% CI 0.28-0.90; P = 0.02), with moderate heterogeneity (I² = 54%) (Figure 4). 

**Figure 4:**
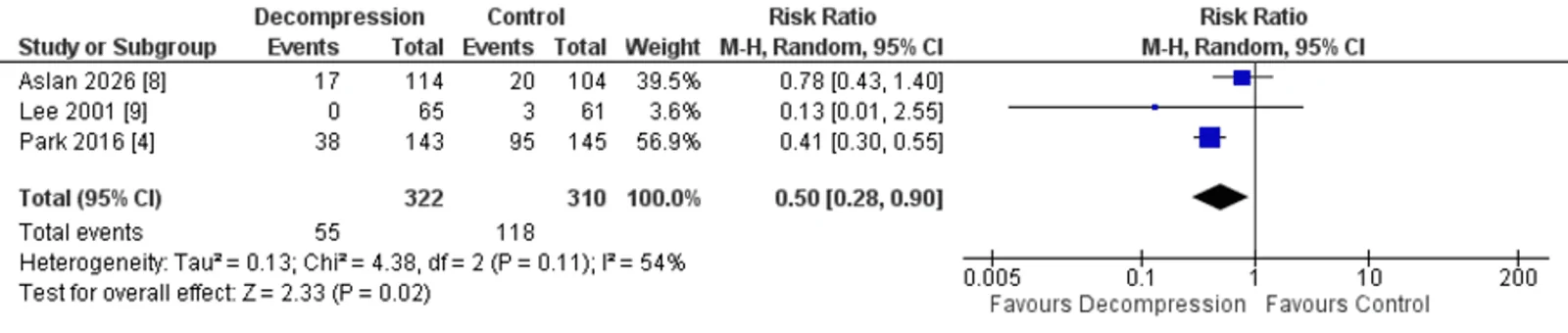
Forest plot: incidence of early post-colonoscopy pain

Incidence of late post-colonoscopy pain (24 hours): No significant difference in the risk of late post-colonoscopy pain was observed between total colonic decompression and control (RR 0.55, 95% CI 0.16 - 1.9, P = 0.35). Pooled studies were homogeneous (P = 0.61, I² = 0%) (Figure 5).

**Figure 5:**
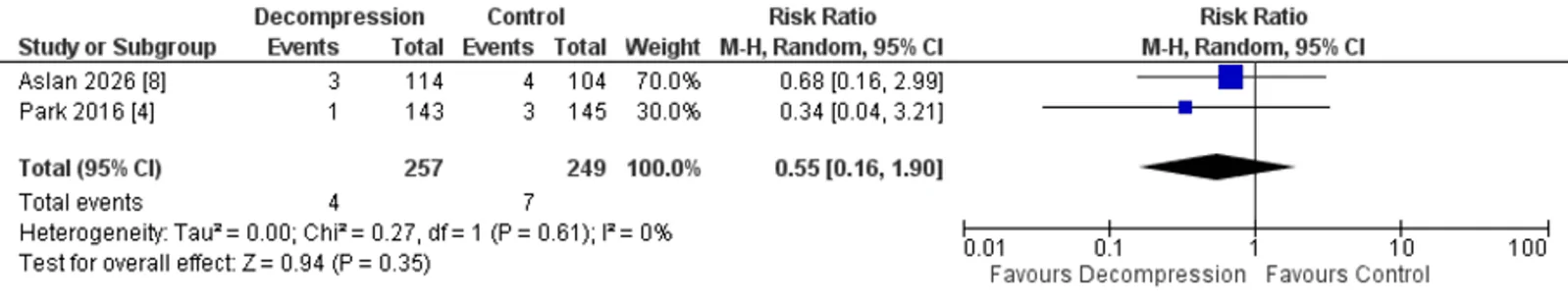
Forest plot incidence of late post-colonoscopy pain

Early post-colonoscopy pain severity (VAS score): The overall mean difference (MD) between the Decompression and the Control groups did not favour either of the two groups (pooled MD = -0.94, 95% CI (-1.97 to 0.09), P = 0.07). Analysis of pooled studies showed substantial statistical heterogeneity (Chi-square P = 0.001; I² = 90%) (Figure 6).

**Figure 6:**
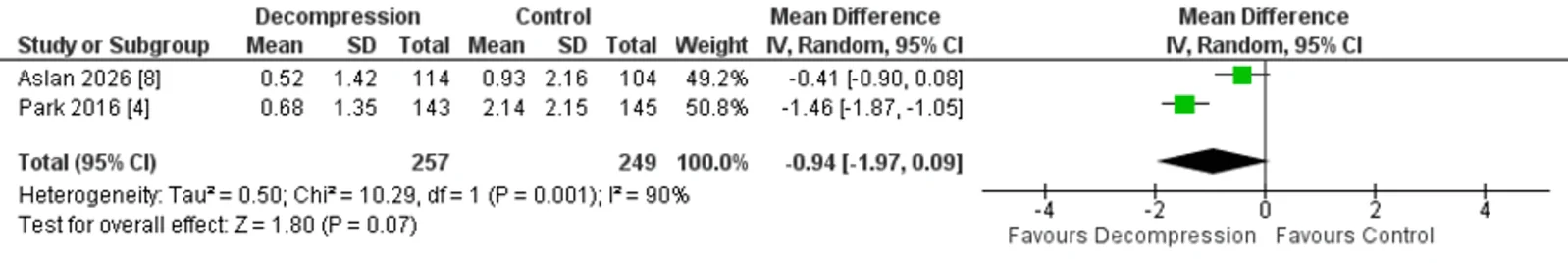
Forest plot: early post-colonoscopy pain severity (VAS score) VAS, visual analog scale

Late post-colonoscopy pain severity (24-hour VAS score): The overall MD between the Decompression and the Control groups did not favour either of the two groups (pooled MD = -0.03, 95% CI (-0.09 to 0.02), P=0.2). Analysis of pooled studies showed no statistical heterogeneity (Chi-square P = 075; I² = 0%) (Figure 7).

**Figure 7:**
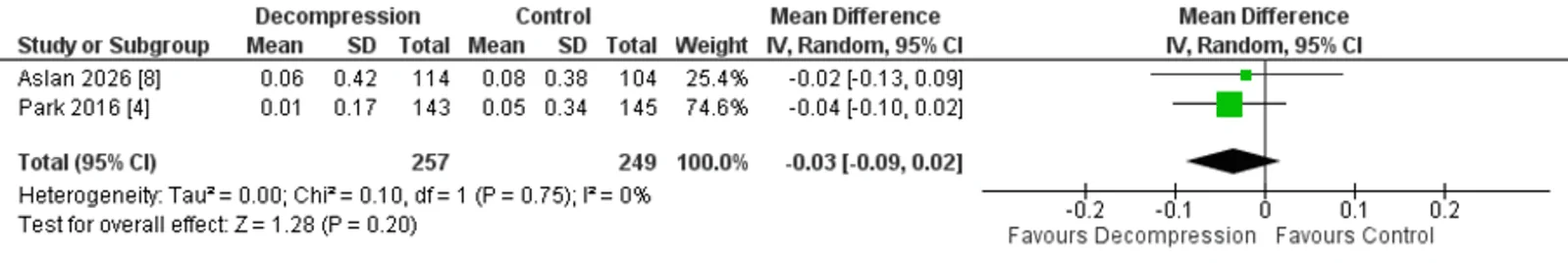
Forest plot: late post-colonoscopy pain severity (24-hour VAS score) VAS, visual analog scale

Discussion

Principal Findings and Clinical Context

The mechanical rationale for total colonic decompression is based on the removal of residual intraluminal air from colonic haustra and flexures before completion of the procedure [4]. By actively aspirating residual gas, decompression may reduce acute colonic distension and thereby contribute to the lower incidence of early post-procedural discomfort observed in some trials [4]. However, the absence of a sustained benefit at 24 hours suggests that the effect of mechanical decompression may be primarily transient, with post-colonoscopy symptoms becoming more similar between intervention and control groups as residual intraluminal gas is subsequently expelled or otherwise cleared.

The potential benefits of decompression should also be considered alongside the procedural demands of scope re-intubation. Re-advancing the colonoscope to proximal colonic segments requires additional procedural manipulation and, particularly under deep sedation, may increase anesthetic requirements [8]. Aslan et al. reported increased total propofol consumption and a higher incidence of transient intraprocedural hypoxemic events in the decompression group, highlighting potential safety considerations associated with the additional procedural time and manipulation [8]. The lack of a significant difference in longer-term pain despite these additional procedural requirements further suggests that the clinical value of routine total colonic decompression may depend on the magnitude of its early symptomatic benefit and the procedural context in which it is performed.

Agreement and Disagreement With Previous Literature

The findings of this meta-analysis capture the conflicting evidence across individual randomized trials while providing a broader quantitative synthesis of the effects of total colonic decompression. Our pooled results align with earlier studies by Lee et al., Park et al., and Sur et al., which reported reductions in acute post-colonoscopy abdominal pain following colonic decompression [9,4,10]. Sur et al. further demonstrated that progressive air evacuation, particularly when reintubation reached more proximal colonic segments such as the hepatic flexure, was associated with lower early post-procedural pain scores [10].

However, our pooled synthesis diverges from these earlier reports regarding overall pain severity and symptom persistence. We found no statistically significant benefit in continuous VAS pain scores during the immediate post-procedural period, nor evidence of sustained symptom reduction at 24 to 48 hours. In this regard, our findings are consistent with the recent trial by Aslan et al., which demonstrated no statistically significant reduction in post-colonoscopy pain at either 30 minutes or 24 hours [8].

These discrepancies across trials may reflect variations in baseline patient characteristics, decompression protocols, procedural characteristics, and, importantly, sedation regimens. Aslan et al. used deep propofol-based sedation, which may have influenced post-procedural gas dynamics and potentially reduced the relative benefit of mechanical air aspiration [8]. Furthermore, Aslan et al. reported that scope reintubation increased total propofol consumption and was associated with a higher incidence of transient intraprocedural hypoxemic events, highlighting potential procedural and safety considerations alongside symptomatic outcomes [8].

Study Strengths and Limitations

Strengths: This is the first systematic review and meta-analysis to quantitatively synthesize randomized controlled evidence on the effect of total colonic decompression after colonoscopy on post-colonoscopy abdominal pain. This study was conducted in accordance with the Cochrane methodological standards and PRISMA 2020 guidelines [11]. Eligibility was restricted to prospective RCTs evaluating total colonic decompression using room air insufflation, ensuring a well-defined intervention and comparator. Quantitative analyses were performed using the inverse-variance and Mantel-Haenszel methods within a random-effects framework, providing pooled estimates of the intervention's effects across clinically relevant outcomes.

Limitations

The primary limitation is the small overall pool of available trials (4 RCTs, 1,037 patients), which restricted statistical power for detailed subgroup analyses and precluded formal assessment of publication bias. Substantial statistical heterogeneity was observed for early pain severity (I² = 90%), likely reflecting methodological variation in sedation depth and scope re-intubation protocols across centers. The included trials also differed in country of conduct, sedation practice, and the precise definition of "early" pain assessment, all of which may have contributed to between-study variability and should be considered when interpreting the pooled estimates. In addition, a formal GRADE assessment of the certainty of evidence was not performed, which may limit the formal assessment of the certainty of the pooled estimates.

## Conclusions

This systematic review and meta-analysis of 4 randomized controlled trials involving 1,037 patients indicates that total colonic decompression reduces the incidence of early post-colonoscopy abdominal pain. However, this benefit does not clearly translate into a significant reduction in pain severity, and no sustained benefit was observed at 24 hours. These findings suggest that the potential benefit of total colonic decompression is primarily limited to reducing the occurrence of early post-procedural pain.

The substantial heterogeneity observed for early pain severity and the limited number of available trials restrict the certainty of these findings. Future larger, multicenter randomized trials should use standardized decompression techniques, sedation protocols, and pain assessment methods to better determine the magnitude and durability of the potential benefit. Until further evidence is available, the use of total colonic decompression should be considered in the context of its potential early symptomatic benefit and the additional procedural requirements involved.
